# Postoperative Inflammatory Marker Surveillance in Colorectal Peritoneal Carcinomatosis

**DOI:** 10.1245/s10434-020-09544-w

**Published:** 2021-03-02

**Authors:** Sasinthiran Thiagarajan, Joey Wee-Shan Tan, Siqin Zhou, Qiu Xuan Tan, Josephine Hendrikson, Wai Har Ng, Gillian Ng, Ying Liu, Grace Hwei Ching Tan, Khee Chee Soo, Melissa Ching Ching Teo, Claramae Shulyn Chia, Chin-Ann Johnny Ong

**Affiliations:** 1 Department of Sarcoma, Peritoneal and Rare Tumours (SPRinT), Division of Surgery and Surgical Oncology, National Cancer Centre Singapore, Singapore, Singapore; 2 Duke-NUS Medical School, Singapore, Singapore; 3 Laboratory of Applied Human Genetics, Division of Medical Sciences, National Cancer Centre Singapore, Singapore, Singapore; 4 Department of Clinical Trials and Epidemiological Sciences, National Cancer Centre Singapore, Singapore, Singapore; 5 Institute of Molecular and Cell Biology, A*STAR Research Entities, Singapore, Singapore; 6 SingHealth Duke-NUS Oncology Academic Clinical Program, Duke-NUS Medical School, Singapore, Singapore

## Abstract

**Background:**

The prognostic significance of inflammatory markers in solid cancers is well-established, albeit with considerable heterogeneity. This study sought to investigate the postoperative inflammatory marker trend in peritoneal carcinomatosis (PC), with a focus on colorectal PC (CPC), and to propose optimal surveillance periods and cutoffs.

**Methods:**

Data were collected from a prospectively maintained database of PC patients treated at the authors’ institution from April 2001 to March 2019. The platelet–lymphocyte ratio (PLR), the neutrophil–lymphocyte ratio (NLR), and the lymphocyte–monocyte ratio (LMR) were collected preoperatively and on postoperative days 0, 1 to 3, 4 to 7, 8 to 21, 22 to 56, and 57 to 90 as averages. Optimal surveillance periods and cutoffs for each marker were determined by maximally selected rank statistics. The Kaplan–Meier method and Cox proportional hazard regression models were used to investigate the association of inflammatory markers with 1-year overall survival (OS) and recurrence-free survival (RFS) using clinicopathologic parameters.

**Results:**

The postoperative inflammatory marker trend and levels did not differ between the patients with and those without hyperthermic intraperitoneal chemotherapy (HIPEC). Low postoperative LMR (days 4–7), high postoperative NLR (days 8–21), and high postoperative PLR (days 22–56) were optimal for prognosticating poor 1-year OS, whereas high postoperative PLR and NLR (days 57–90) and low postoperative LMR (days 8–21) were associated with poor 1-year RFS. A composite score of these three markers was prognostic for OS in CPC.

**Conclusions:**

The reported cutoffs should be validated in a larger population of CPC patients. Future studies should account for the inflammatory response profile when selecting appropriate surveillance periods.

**Supplementary Information:**

The online version of this article (10.1245/s10434-020-09544-w) contains supplementary material, which is available to authorized users.

Inflammation is a hallmark of cancer,^1^ and its role in oncogenesis and cancer progression is well-established.^2^–^5^ Inflammatory marker ratios such as the platelet–lymphocyte ratio (PLR), the neutrophil–lymphocyte ratio (NLR), and the lymphocyte–monocyte ratio (LMR) have been widely studied for this purpose in solid tumors.^6^–^8^

However, substantial heterogeneity exists between these studies, as highlighted in several meta-analysis reports,^8^,^9^ and this heterogeneity limits translatability to clinical practice. First, there is no consensus on a cutoff for each marker, either pre- or postoperatively. Second, the studies differ significantly in the method whereby appropriate cutoffs are determined, whereas other studies report the significance of these markers as continuous variables without using a cutoff or methods to derive cutoffs specific to the study cohort that may not be generalizable. Third, each inflammatory marker may or may not be prognostic for each primary tumor type and has different cutoffs.

Although fewer studies have investigated postoperative inflammatory markers, the studies differ greatly in the specific postoperative period during which the inflammatory markers were surveyed. Studies on the postoperative trends of these inflammatory markers also suggest that different cutoffs should be used for different postoperative time points.^10^,^11^

Peritoneal carcinomatosis (PC) is a common type of metastasis originating from a variety of intraabdominal organs. For a carefully selected group of patients with PC, cytoreductive surgery (CRS) and hyperthermic intraperitoneal chemotherapy (HIPEC) can be used to improve patient outcomes. Reports have suggested that postoperative inflammatory cell response is affected by HIPEC treatment and that such trends can be used to perdict postoperative infective complications.^12^,^13^ However, to our knowledge, it is unclear whether inflammatory markers follow a different postoperative course for patients who underwent HIPEC than for those who did not. Therefore, although a large volume of studies on this topic has been published, their translatability to clinical practice has been limited.

With this in mind, we sought to investigate the prognositic utility of postoperative inflammatory marker surveillence for patients with PC in a large tertiary institution in Asia. In doing so, we aimed to determine the optimal period for surveillence and to propose a cutoff for these markers that can be validated in a larger multicenter patient population.

## Materials and Methods

This study was approved by the SingHealth Centralised Institutional Review Board (IRB reference: 2018/2638) and conducted in compliance with all applicable SingHealth institutional policies and regulations. Demographic and clinicopathologic data for this study were collected from a prospectively maintained database of all patients with PC treated at the National Cancer Centre Singapore (NCCS) from April 2001 to March 2019.

The study constructed PLR, NLR, and LMR by taking the ratios of the absolute counts of the respective components of the full blood count panel, where available, for each patient before (up to 1 week before the date of the procedure) and after surgery during 90 days. To determine the ideal postoperative period for surveillance, averages of these ratios were taken preoperatively and on postoperative days 0, 1 to 3, 4 to 7, 8 to 21, 22 to 56, and 57 to 90. These surveillance periods were determined based on a previous study of colorectal cancer patients^11^ and supported by our observation of the general trend of the host immune response during the postoperative period (Fig. S1).

Overall survival (OS) was defined as the period from the date of surgery to the date of death from any cause or the last follow-up visit, and recurrence-free survival (RFS) was defined as the period from the date of surgery to the date of disease recurrence at any site. Cancer staging was based on routine postoperative histopathologic analysis and clinical assessment according to the American Joint Committee on Cancer (AJCC) staging manual.

### Statistical Analyses

The Kruskal–Wallis test was used to compare the distribution of inflammatory marker ratios across different tumor subtypes, and the paired-samples *t* test was used to compare postoperative inflammatory marker levels with preoperative levels to determine when they returned to preoperative levels. The independent-samples Mann–Whitney *U* test was performed on inflammatory markers and ratios to compare the patients who underwent HIPEC with those who did not. Cutoffs for PLR, NLR, and LMR at each surveillance period were determined using the maximally selected rank statistics^14^,^15^ on the R package Maxstat with the Horton and Lausen (HL) *p* value approximation method.^16^

The patients were subsequently dichotomized into “high” and “low” groups based on the cutoffs at each time point. The 1-year OS and RFS were analyzed using the Kaplan–Meier method for each inflammatory marker at each period, and the log-rank test was used to determine the hazard ratio. A composite score of the different markers across time was constructed, and the prognostic significance was determined by the Kaplan–Meier method for 1-year OS and RFS.

Cox proportional hazard regression models were used to investigate whether an association existed between the composite score and the 1-year OS in both the uni- and multivariate analyses with clinicopathologic parameters. Variables with a *p* value of 0.1 or lower in the univariate analysis were progressed to a multivariate analysis using backward logistic regression.

All analyses were performed using SPSS software version 25 (SPSS Inc., Chicago, IL, USA) and R (version 3.6.2, open source). A two-sided *p* value lower than 0.05 was considered to be statistically significant.

## Results

### Patient Characteristics

For this study, 436 patients with PC who underwent surgery at our center between April 2001 and March 2019 were recruited with informed consent. Of these 436 patients, 331 (75.9%) underwent HIPEC. A majority of the recruited patients (*n* = 161, 36.9%) had colorectal PC, whereas 121 patients (22.8%) had ovarian PC, 111 patients (25.5%) had appendiceal PC, 26 patients (6%) had primary peritoneal cancer, and 17 patients (3.9%) had mesothelioma (Fig. S2).

In a subpopulation of patients with colorectal PC at our center (*n* = 161), 84.5% (*n* = 136) had CRS and 80.7% (*n* = 130) had HIPEC. The median age of the patients at the time of treatment was 57 years (range, 47–65 years) (Table 1). The majority of the patients (70.2%, *n* = 113) were older than 50 years. The patients were predominantly female (59%, *n* = 95) and Chinese (82%, *n* = 132) with an Eastern Cooperative Oncology Group (ECOG) score of either 0 (80.7%, *n* = 130) or 1 (9.3%, *n* = 15). Whereas 88.8% of the patients had metachronous colorectal PC (CPC), 11.2% had synchronous CPC. The median peritoneal cancer index (PCI) score was 6 (range, 3–12.5), and the completeness of cytoreduction (CC) score was mainly 0 (81.4%, *n* = 131). The median hospital stay was 12 days (range, 9–15.5 days). The median OS was 18 months (range, 10–33) and the median RFS was 11 months (range, 6–19 months). The distribution of tumor-node-metastasis (TNM) stages and histologic subtypes are reported in Table 1.

**Table 1:** Demographic and clinicopathologic characteristics of patients with colorectal peritoneal carcinomatosis (CPC) (*n* = 161)

Variable		*n* (%)
No. of CRS		136 (84.5)
No. of HIPEC		130 (80.7)
Median age at CRS/CRS-HIPEC: years (IQR)	≤50 >50	57 (47–65) 48 (29.8) 113 (70.2)
Gender	Male Female	66 (41.0) 95 (59.0)
Race	Chinese Malay Indian Others	132 (82.0) 6 (3.7) 3 (1.9) 20 (12.4)
ECOG status	0 1	130 (80.7) 15 (9.3)
Median hospital stay: days (IQR)		12.00 (9.00–15.50)
Histology	Adenocarcinoma Mucinous adenocarcinoma Mucinous adenocarcinoma with signet-ring cell Signet-ring cell Tubulovillous adenoma Unknown	109 (67.7) 35 (21.7) 3 (1.9) 6 (3.7) 2 (1.2) 6 (3.7)
Tumor grade	1 2 3 Unknown	1 (0.6) 60 (37.3) 7 (4.3) 75 (46.6)
T stage	X 1 2 3 3B 4 4A 4B Unknown	4 (2.5) 1 (0.6) 3 (1.9) 42 (26.1) 1 (0.6) 31 (19.3) 36 (22.4) 22 (13.7) 12 (7.5)
N stage	0 1 1A 1B 1C 2 2A 2B X Unknown	45 (28.0) 20 (12.4) 8 (5.0) 11 (6.8) 3 (1.9) 16 (9.9) 16 (9.9) 16 (9.9) 5 (3.1) 12 (7.5)
M stage	0 1 X Unknown	77 (47.8) 59 (36.6) 1 (0.6) 14 (8.7)
Synchronous CPC Metachronous CPC		18 (11.2) 143 (88.8)
Had neoadjuvant therapy		9 (5.6)
Had adjuvant therapy		101 (62.7)
Complications (Clavien-Dindo)	Grade 1 Grade 2 Grade 3 Grade 4 Grade 5	15 (9.3) 31 (19.3) 17 (10.6) 1 (0.6) 0 (0.0)
Comorbidities	Hypertension Diabetes Hyperlipidaemia Ischemic heart disease COPD Asthma Other malignancy Others None	47 (29.2) 21 (13.0) 32 (19.9) 3 (1.9) 1 (0.6) 4 (2.5) 6 (3.7) 64 (39.8) 47 (29.2)
Median PCI score (IQR)		6.00 (3.00–12.50)
CC score	0 1 2 3 Unknown	131 (81.4) 3 (1.9) 1 (0.6) 7 (4.3) 19 (11.8)
OS: months (IQR)		18 (10–33)
RFS: months (IQR)		11 (6–19)

*CRS* cytoreductive surgery, *HIPEC* hyperthermic intraperitoneal chemotherapy, *IQR* interquartile range, *ECOG* Eastern Cooperative Oncology Group, *COPD* chronic obstructive pulmonary disease, *PCI* peritoneal carcinomatosis index, *CC* completeness of cytoreduction, *OS* overall survival, *RFS* recurrence free survival

### PLR, NLR, and LMR Trends

A comparison of ratios surveyed at different time points and preoperative levels is presented in Table S1. Both PLR and NLR returned to preoperative levels between postoperative days 57 and 90, whereas LMR returned to preoperative levels between days 22 and 56.

Although the general profiles of PLR, NLR, and LMR for each primary tumor type were similar for all 436 patients in the study (Fig. S3), the distribution of ratio levels differed significantly during various time points (*p* < 0.001 for PLR; *p* = 0.040 for NLR; and *p* = 0.001 for LMR). Notably, primary tumor subtypes differed in the duration of postoperative inflammatory response, as evidenced by a longer duration for elevated NLR in mesothelioma and appendiceal origin patients (up to postoperative days 8 to 21), compared with other primary tumor types (up to postoperative days 1 to 3) (Fig. S3).

We were unable to get a unified conclusion from our cohort on the effect that different treatment regimens had on the inflammatory marker ratios due to the considerable variation in treatment regimens among our patients (Table S2). Furthermore, in comparing the levels of inflammatory cell levels at different time points postoperatively, we found no significant difference between those who underwent HIPEC and those who did not, except for the level of all leukocytes and neutrophils on days 8 (*p* = 0.02) to 21 (*p* = 0.018) and the platelet levels on days 57 to 90 (*p* = 0.024) (Fig. S4). However, the postoperative course of inflammatory marker ratios (PLR, NLR, and LMR) did not differ between the patients who underwent HIPEC and those who did not.

**Fig. 1:**
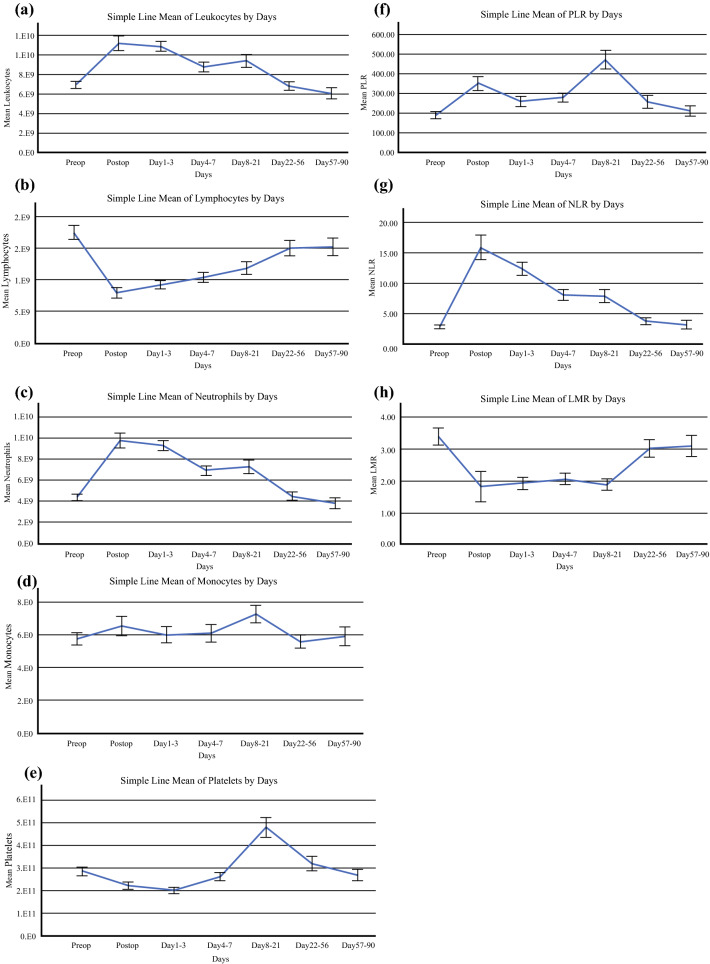
Trend of the platelet–lymphocyte ratio (PLR), neutrophil–lymphocyte ratio (NLR), lymphocyte–monocyte ratio (LMR), and the constituent inflammatory cell levels during the pre- and postoperative periods surveyed (mean ± two standard deviations). The PLR increased within 24 h after surgery, driven by a gradual and slight decrease in platelet levels, with a sharp decline in lymphocyte levels (**f, b, e**). Whereas the lymphocyte levels continued to increase after this period to about 22 to 56 days, the platelet levels continued to fall until days 4 to 7, when a delayed thrombocytosis occurred up to days 8 to 21, resulting in the PLR ratios reaching a peak during this period, followed by a decrease to baseline levels after 22 to 56 days, primarily driven by a decreasing platelet count in the background of rising lymphocyte counts. The NLR rose sharply within 24 h after surgery due to a significant increase in neutrophil levels and a significant decrease in lymphocyte levels immediately after surgery (**g, b, c**). Subsequently, the NLR gradually declined to preoperative levels between days 22 and 90 as lymphocyte levels gradually increased while neutrophil levels decreased during this period. The LMR dropped sharply within 24 h after surgery and stabilized at this level up to days 8 to 21, after which it increased back to preoperative levels (**h**). This trend was primarily driven by a sharp drop in lymphocytes immediately after surgery, with a subsequent gradual increase to preoperative levels and two periods of transient increase in monocyte levels: within 24 h after surgery and a more significant increase during postoperative days 8 to 21 (**b, d**)

Given the variation in inflammatory marker trends between primary tumor subtypes and the differences observed in the literature with regard to cutoffs for different primary solid tumor types, we decided to focus on the subset of patients with CPC because it is the most common primary tumor type. Furthermore, we found no difference in levels of the inflammatory markers between the patients who underwent HIPEC and those who did not (Fig. S4). We therefore included both groups of patients in our subsequent analysis. The trends of PLR, NLR, and LMR and the corresponding profiles of the constituent inflammatory cells over the pre- and postoperative periods surveyed are presented in Fig. 1. These trends were similar to the profile of inflammatory response observed in the data pooled for all 436 patients across primary tumor subtypes (Figs. S1 and S3).

### Optimal Cutoff and Postoperative Surveillance Period for 1-Year OS

Subjecting our subpopulation of 161 CPC patients to maximally selected rank statistics analyses, we determined the optimal cutoffs for each surveillance period (Table 2). An elevated PLR was associated with a poor survival at all time points surveyed, and the best hazard ratio (HR) for 1-year OS was obtained during postoperative days 22 to 56 (HR, 5.629; 95% confidence interval [CI] 3.087–10.264; *p* < 0.001), with a cutoff of 369.65. Whereas preoperative NLR was not prognostic for 1-year OS, a low postoperative NLR within 24 h at a cutoff of 6.33 (HR, 0.304; 95% CI, 0.175–0.526; *p* < 0.001) and a high postoperative NLR beyond 24 h to 90 days were associated with a poor 1-year OS. The best HR for a poor 1-year OS associated with a high postoperative NLR was during postoperative days 8 to 21 (HR, 3.638; 95% CI, 1.894–6.988; *p* < 0.001), with a cutoff of 13.26. A high early postoperative LMR within 24 h (HR, 2.403; 95% CI, 1.419–4.070; *p* = 0.001) at a cutoff of 2.32, a low preoperative LMR (HR, 0.502; 95% CI, 0.312–0.807; *p* = 0.004), and a low postoperative LMR beyond 4 days were associated with a poor 1-year OS. The best HR ratio for a poor 1-year OS associated with a low postoperative LMR was during postoperative days 4 to 7 (HR, 0.172; 95% CI, 0.091–0.325; *p* < 0.001) at a cut-off of 0.93. When the LMR was surveyed between postoperative days 1 to 3, it was not prognostic for 1-year OS.

**Table 2:** Cutoff determination for PLR, NLR, and LMR using maximally selected rank statistics

Marker		1-Year overall survival					1-Year recurrence free survival				
		Cutoff		HR	95% CI	*p* value	Cutoff		HR	95% CI	*p* value
PLR	Preop	245.93	Below: 131 Above: 30	2.896	1.697–4.943	< 0.001	111.73	Below: 36 Above: 125	**1.447**	**0.932–2.245**	**0.100**
	Postop < 24 h	512.90	Below: 128 Above: 26	2.702	1.523–4.792	0.001	155.56	Below: 21 Above: 133	**0.637**	**0.388–1.047**	**0.075**
	Post-op days 1–3	255.40	Below: 103 Above: 53	2.250	1.412–3.586	0.001	389.17	Below: 137 Above: 19	**1.436**	**0.816–2.527**	**0.209**
	Postop days 4–7	288.15	Below: 92 Above: 49	3.081	1.891–5.021	< 0.001	285.37	Below: 90 Above: 51	1.576	1.051–2.363	0.028
	Postop days 8–21	546.36	Below: 77 Above: 33	2.341	1.374–3.990	0.002	251.83	Below: 19 Above: 91	**1.568**	**0.879–2.798**	**0.128**
	Postop days 22–56	369.65	Below: 92 Above: 20	5.629	3.087–10.264	< 0.001	200.44	Below: 57 Above: 55	1.751	1.131–2.711	0.012
	Postop days 57–90	141.79	Below: 31 Above: 67	4.788	2.021–11.341	< 0.001	101.80	Below: 16 Above: 82	7.127	2.525–20.116	<0.001
NLR	Preop	2.99	Below: 108 Above: 53	**1.553**	**0.973–2.480**	**0.065**	1.41	Below: 15 Above: 146	**1.449**	**0.750–2.797**	**0.270**
	Postop < 24 h	6.33	Below: 22 Above: 132	0.304	0.175–0.526	<0.001	7.20	Below: 27 Above: 127	0.453	0.282–0.728	0.001
	Postop days 1–3	18.71	Below: 135 Above: 21	2.401	1.405–4.104	0.001	14.83	Below: 118 Above: 38	**0.711**	**0.461–1.096**	**0.122**
	Postop days 4–7	7.99	Below: 89 Above: 52	3.298	2.022–5.379	<0.001	7.73	Below: 84 Above: 57	1.785	1.202–2.652	0.004
	Postop days 8–21	13.26	Below: 96 Above: 14	3.638	1.894–6.988	<0.001	5.83	Below: 50 Above: 60	1.601	1.030–2.490	0.037
	Postop days 22–56	2.76	Below: 53 Above: 59	2.732	1.489–5.015	0.001	2.76	Below: 53 Above: 59	1.864	1.188–2.925	0.007
	Postop days 57–90	3.48	Below: 74 Above: 24	2.618	1.465–4.679	0.001	1.57	Below: 28 Above: 70	2.416	1.360–4.289	0.003
LMR	Preop	2.86	Below: 63 Above: 98	0.502	0.312–0.807	0.004	4.59	Below: 130 Above: 31	**0.794**	**0.506–1.245**	**0.315**
	Postop < 24 h	2.32	Below: 130 Above: 24	2.403	1.419–4.070	0.001	2.87	Below: 137 Above: 17	**1.419**	**0.806–2.498**	**0.226**
	Postop days 1–3	3.03	Below: 138 Above: 18	**0.485**	**0.217–1.080**	**0.076**	2.68	Below: 133 Above: 23	0.514	0.290–0.909	0.022
	Postop days 4–7	0.93	Below: 14 Above: 127	0.172	0.091–0.325	<0.001	3.09	Below: 126 Above: 15	0.312	0.143–0.680	0.003
	Postop days 8–21	0.91	Below: 14 Above: 96	0.327	0.162–0.659	0.002	2.88	Below: 97 Above: 13	0.264	0.105–0.663	0.005
	Postop days 22–56	1.93	Below: 30 Above: 82	0.348	0.201–0.602	<0.001	2.37	Below: 43 Above: 69	0.544	0.347–0.851	0.008
	Postop days 57–90	1.78	Below: 19 Above: 79	0.503	0.266–0.950	0.034	1.56	Below: 11 Above: 87	0.349	0.162–0.751	0.007

Values in bold indicate *p* > 0.05

*HR* hazard ratio, *CI* confidence interval, *Preop* preoperative, *Postop*, postoperative, *PLR* platelet–lymphotycte ratio, *NLR* neutrophil–lymphocyte ratio, *LMR* lymphocyte–monocyte ratio

### Optimal Cutoff and Postoperative Surveillance Period for 1-Year RFS

Preoperative PLR was not prognostic for 1-year RFS. An elevated postoperative PLR was associated with a poor survival at 4 to 7 days and at 22 to 90 days, whereas the best HR for 1-year RFS was obtained during postoperative days 57 to 90 (HR, 7.127; 95% CI, 2.525–20.116; *p* < 0.001), with a cutoff of 101.80. Whereas pre- and postoperative NLR from days 1 to 3 were not prognostic for 1-year RFS, a low postoperative NLR within 24 h at a cutoff of 7.20 (HR, 0.453; 95% CI, 0.282–0.728; *p* = 0.001) and a high postoperative NLR beyond 4 to 90 days were associated with a poor 1-year RFS. The best HR ratio for a poor 1-year RFS associated with a high postoperative NLR was during postoperative days 57 to 90 (HR, 2.416; 95% CI, 1.360–4.289; *p* = 0.003), with a cutoff of 1.57. Preoperative LMR and LMR surveyed within postoperative 24 h were not prognostic for 1-year RFS. A low postoperative LMR beyond 24 h was associated with a poor 1-year RFS. The best HR for a poor 1-year RFS associated with a low postoperative LMR was during days 8 to 21 (HR, 0.264; 95% CI, 0.105–0.663; *p* = 0.005) at a cutoff of 2.88.

### Composite Score for OS and RFS

**Fig. 2:**
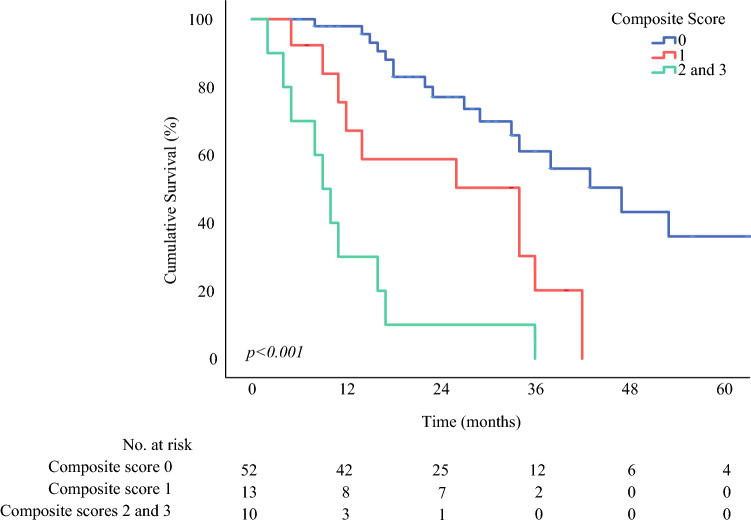
Kaplan-Meier survival analysis for a 1-year OS composite LMR score (4–7 days), NLR score (8–21 days), and PLR score (22–56 days) (*p* < 0.001)

A composite score for 1-year OS and RFS was computed based on the aforementioned cutoffs and optimal surveillance periods. A low postoperative LMR on days 4 to 7, a high postoperative NLR on days 8 to 21, and a high postoperative PLR on days 22 to 56, were assigned values of 1 for a 1-year OS composite score. A high PLR and NLR on postoperative days 57 to 90 and a low LMR on postoperative days 8 to 21 were assigned values of 1 for a 1-year RFS composite score. The composite scores were subjected to Kaplan-Meir survival analyses (Fig. 2). Scores of 0, 1, and a combination of 2 and 3 showed a significant difference in 1-year OS (*p* < 0.001) but not in 1-year RFS.

### Uni- and Multivariate Analyses

The composite score for 1-year OS was subjected to uni- and multivariate analyses with other clinicopathologic and demographic patient characteristics. In the univariate analysis, race, cytoreductive surgery (CRS), duration of peritonectomy, completeness of cytoreduction (CC) score, intraoperative complications, and the composite score were prognostic for 1-year OS at a *p* value lower than 0.1 (Table 3). In the multivariate analysis, only the composite score, race, and intraoperative complications were prognostic for 1-year OS at a *p* value lower than 0.05 (Table 4).

**Table 3:** Univariate overall survival analysis

Marker/variable	HR	95% CI	*p* value
Race			
Chinese	Ref		**0.057**
Malay	1.127	0.271 to 4.675	0.870
Indian	<0.001	<0.001 to < 0.001	0.977
Others	2.486	1.295 to 4.770	**0.006**
CRS performed	0.246	0.138 to 0.438	**<0.001**
Duration of peritonectomy	1.003	1.001 to 1.005	**0.008**
CC score			
0	Ref		**<0.001**
1	3.741	0.892 to 15.690	0.071
2	<0.001	<0.001 to < 0.001	0.980
3	9.735	4.202 to 22.553	**<0.001**
Intraoperative complications	4.498	1.706 to 11.856	**0.002**
Composite score			
0	Ref		**0.004**
1	1.682	0.527 to 0.5363	0.380
2 and 3	5.219	1.980 to 13.755	**0.001**

Values in bold indicate *p* < 0.1

*HR* hazard ratio, *CI* confidence interval, *CRS* cytoreductive surgery, *CC* completeness of cytoreduction

**Table 4:** Multivariate overall survival analysis

Marker/variable	HR	95% CI	*p* value
Composite score			
0	Ref		**<0.001**
1	0.258	0.014–4.825	0.365
2 and 3	30.912	6.417–148.916	**<0.001**
Race			
Chinese	Ref		**0.019**
Malay	11.224	0.980–128.506	0.052
Others	8.530	1.607–45.286	**0.012**
Intraoperative complications	9.919	1.106–88.934	**0.040**

Values in bold indicate *p* < 0.05

*HR* hazard ratio, *CI* confidence interval

## Discussion

The current study addressed an unanswered question on the utility of inflammatory markers for PC patients. The results showed that inflammatory markers measured at specific time points during postoperative follow-up evaluation may allow us to identify patients requiring closer monitoring for disease recurrence or poor survival. Specifically, a low postoperative LMR (days 4–7) at a cutoff of 0.93, a high postoperative NLR (days 8–21) at a cutoff of 13.26, and a high postoperative PLR (days 22–56) at a cutoff of 369.65 were determined to provide the best prognostic information for poor 1-year OS. A composite score of these three markers was also prognostic for 1-year OS in the uni- and multivariate analyses.

We also found that a high postoperative PLR and NLR (days 57–90) at a cutoff of 101.80 and 1.57, respectively, and a low postoperative LMR (days 8–21) at a cutoff of 2.88 were associated with a poor 1-year RFS. However, a composite score of these markers was not prognostic for 1-year RFS.

Although the periods and cutoffs identified do not agree with a previous study on primary colorectal cancer,^11^ this difference may be explained by the fact that inflammatory markers in PC may have a prognostic difference from that of primary tumors, as previously suggested.^17^ Therefore, further research into the prognostic role of inflammatory markers in peritoneal metastatic cancers is warranted because up to 60% of ovarian, 50% of pancreatic, and 32% of colon cancers will develop PC, with a significant impact on patients’ quality of life (e.g., abdominal distension and intestinal obstruction).^18^–^20^

Despite advances in aggressive treatment strategies, including a combination of CRS and HIPEC, long-term survival remains low, with meta-analyses citing a median OS of 35.3 months, a median RFS of 2 years, and a 5-year average OS rate of 40% for patients with CPC.^21^,^22^ Given the poor patient outcomes, further research in this area could potentially lead to enhanced surveillence and early intervention for patients at higher risk of mortality and disease recurrence.

In this report, we characterized the trends of the inflammatory marker ratios and the constituent blood cell levels during 90 days after surgery for all 436 patients and specifically for 161 patients with CPC. Given that we found differences between primary tumor subtypes in PC, our proposed cutoffs and surveillance periods are perhaps generalizable only to patients with CPC. However, the general trend of changes in the postoperative inflammatory cell and ratio levels appears consistent across primary tumor subtypes, with the exception of NLR, despite the differences between tumor subtypes. Therefore, it will be interesting to study whether the trends are similar across other cancer types, including non-metastatic cancers, and whether any variation in the trend could be prognostic of an adverse outcome.

Additionally, we also report no difference in the postoperative course or levels of the inflammatory markers between the patients who underwent HIPEC and those who did not. To our knowledge, this is the first report to compare the postoperative trend of inflammatory response between HIPEC and non-HIPEC patients during a 3-month period.

Prior studies have reported that high postoperative monocyte counts are associated with poor survival for lung cancer patients^23^,^24^ within 4 days after surgery and for esophageal cancer patients 1 week after surgery. Although we similarly found that a high LMR measured within 24 h postoperatively was associated with a poorer prognosis, we also found that a high postoperative LMR beyond 1 day after surgery was associated with a better prognosis. Although this difference may be attributable to differences in primary tumor biology and surveillance periods, it is critical to understand whether the change from a poor prognosis to a good prognosis associated with a high monocyte count could be explained by temporal differences in the proportion of M1 proinflammatory and antitumor macrophages to M2 protumorigenic macrophages.^25^–^27^ This could be studied by the use of fluorescence-activated cell-sorting (FACS) analysis of various markers to decipher the M1 and M2 macrophage populations.^28^

Early postoperative changes in the host immune macroenvironment may be important in determining recurrence or survival, and studying the mechanisms underlying differences between patients may provide insights into potential immunotherapeutic strategies. Furthermore, given the temporal changes in the levels of postoperative inflammatory marker levels and the differences in prognostic significance at different time points that we have reported, future studies on the prognostic value of such markers should account for this variation when surveillance periods are selected so that studies are comparable and results can be interpreted with more certainty.

Although the findings of this study are novel and provide critical information on the utility of inflammatory markers in the prognosis of HIPEC patients, the study had a few limitations. We sought to investigate the prognostic potential of inflammatory markers in peritoneal cancers using a cohort of 436 patients, but we had to limit our study to a smaller subpopulation of 161 CPC patients after learning that the primary tumor types had an influence on the postoperative longitudinal profile of inflammatory markers as well as the cutoffs. Furthermore, given that the patients included in this study were recruited during an 18-year period with varied treatment regimens, we were not able to account for changes in treatment strategies for primary tumor subtypes and HIPEC protocols over the years. Nevertheless, this was one of the largest studies to examine the different levels of inflammatory markers at different time points in CPC, one of the most common peritoneal diseases,. Additionally, we validated our results in the largest cohort of CPC patients who underwent HIPEC to date, although it would be noteworthy to see whether this holds true in future prospective cohorts, as well as in cohorts of varying histologies.

Although these blood biomarkers provide a simple, cost-effective, and readily reproducible prognostic tool for patient management, their clinical translatability has been limited by the heterogeneity of previous studies, the lack of consensus on optimal periods for surveillance, and ideal cutoffs validated in large multicenter cohorts. As such, our study, one of the few studies with an aim to understand this, provides much needed value to the current literature. We await future studies to validate our results.

## Conclusion

In conclusion, postoperative surveillance of inflammatory markers serially may allow for prognostication of poor disease outcome and allow for identification of patients requiring more frequent follow-up assessments or early intervention. Future studies should focus on validating the proposed cutoffs and surveillance period in large multi-center studies of the same cancer type.

## Supplementary Information

Supplemetary Figure S1. Profile of immune cell response in patients across time (i.e. from pre-operation to postoperative day 90). Supplementary Figure S2: Flowchart illustrating study population. Supplementary Figure S3: Trend of PLR, NLR and LMR in patients across time (i.e. from pre-operation to postoperative day 90) by primary tumour type. Supplementary Figure S4: Comparison of the profile of immune cell response in all peritoneal carcinomatosis patients across time (i.e. from pre-operation topostoperative day 90) based on whether they underwent HIPEC (n = 331) or no HIPEC (n = 39)*denotes significance at *p* < 0.05 for Mann-Whitney U Test. Supplementary material 1 (DOCX 989 kb)
