# Supplementary material for: Postoperative Inflammatory Marker Surveillance in Colorectal Peritoneal Carcinomatosis
Source: Ann Surg Oncol. 2021 Mar 2;28(11):6625–35. doi: 10.1245/s10434-020-09544-w (PMC8460570; doi:10.1245/s10434-020-09544-w)
Supplement: Supplementary file 1 — Supplemetary Figure S1. Profile of immune cell response in patients across time (i.e. from pre-operation to postoperative day 90). Supplementary Figure S2: Flowchart illustrating study population. Supplementary Figure S3: Trend of PLR, NLR and LMR in patients across time (i.e. from pre-operation to postoperative day 90) by primary tumour type. Supplementary Figure S4: Comparison of the profile of immune cell response in all peritoneal carcinomatosis patients across time (i.e. from pre-operation topostoperative day 90) based on whether they underwent HIPEC (n = 331) or no HIPEC (n = 39)*denotes significance at p < 0.05 for Mann-Whitney U Test. Supplementary material 1 (DOCX 989 kb) [file 10434_2020_9544_MOESM1_ESM.docx]

**Postoperative inflammatory marker surveillance in colorectal peritoneal carcinomatosis**

Running Head: Postop CPC inflammatory marker surveillance

Sasinthiran Thiagarajan^1,2^*, BSc

Joey Wee-Shan Tan^1,3^*, MSc

Siqin Zhou^4^, MSc

Qiu Xuan Tan^1,3^, BSc

Josephine Hendrikson^1,3^, BSc

Wai Har Ng^1,3^, MSc

Gillian Ng^1,3^, Diploma

Ying Liu^1,3^, PhD

Grace Hwei Ching Tan^1^, MBBS, MMed, FRCS

Khee Chee Soo^1,6^, MBBS, MD, FRACS, FACS

Melissa Ching Ching Teo^1,6^, MBBS, MMed, FRCS, MPH

Claramae Shulyn Chia^1,6^, MBBS, MMed, FRCS

Chin-Ann Johnny Ong^1,3,5,6^**, MBBS, MMed, FRCS, PhD

^1^ Department of Sarcoma, Peritoneal and Rare Tumours (SPRinT), Division of Surgery and Surgical Oncology, National Cancer Centre Singapore, Singapore

^2^ Duke-NUS Medical School, Singapore

^3^ Laboratory of Applied Human Genetics, Division of Medical Sciences, National Cancer Centre Singapore, Singapore

^4^ Department of Clinical Trials & Epidemiological Sciences, National Cancer Centre Singapore, Singapore

^5^ Institute of Molecular and Cell Biology, A*STAR Research Entities, Singapore

^6^ SingHealth Duke-NUS Oncology Academic Clinical Program, Duke-NUS Medical School, Singapore, Singapore

* Equal contribution

** Corresponding author

**Correspondence to:**

Assistant Professor Chin-Ann Johnny Ong

Consultant, Department of Sarcoma, Peritoneal and Rare Tumours (SPRinT), Division of Surgery and Surgical Oncology, National Cancer Centre Singapore

Principal Investigator, Laboratory of Applied Human Genetics, Division of Medical Sciences, National Cancer Centre Singapore

Address: 11 Hospital Crescent Singapore 169610

Email address: [johnny.ong.c.a@singhealth.com.sg](mailto:johnny.ong.c.a@singhealth.com.sg)

Tel no.: +65 64368318

Fax no.: +65 62257559

**Disclosure:** NA

**Supplementary Table S1.** Comparison of postoperative levels of PLR, NLR and LMR to preoperative levels.

|  | | **n** | **Mean** | **Std. Deviation** | ***p*-value** |
| --- | --- | --- | --- | --- | --- |
| PLR | Pre_PLR | 154 | 187·3459 | 121·67427 | <0·001 |
|  | Post_PLR | 154 | 349·5737 | 214·15793 |  |
|  | Pre_PLR | 156 | 185·3791 | 119·88244 | <0·001 |
|  | PLR_1_3 | 156 | 257·6505 | 156·90973 |  |
|  | Pre_PLR | 141 | 182·8699 | 122·31300 | <0·001 |
|  | PLR_4_7 | 141 | 278·0572 | 132·87895 |  |
|  | Pre_PLR | 110 | 194·5539 | 131·99190 | <0·001 |
|  | PLR_8_21 | 110 | 470·0503 | 254·30959 |  |
|  | Pre_PLR | 112 | 199·1694 | 132·94043 | 0·003 |
|  | PLR_22_56 | 112 | 254·8966 | 174·83580 |  |
|  | **Pre_PLR** | **98** | **197·8894** | **126·40405** | **0·487** |
|  | **PLR_57_90** | **98** | **209·4319** | **132·18706** |  |
| NLR | Pre_NLR | 154 | 2·8635 | 1·84132 | <0·001 |
|  | Post_NLR | 154 | 15·9165 | 12·43156 |  |
|  | Pre_NLR | 156 | 2·8909 | 1·91296 | <0·001 |
|  | NLR_1_3 | 156 | 12·3954 | 6·75174 |  |
|  | Pre_NLR | 141 | 2·7911 | 1·75874 | <0·001 |
|  | NLR_4_7 | 141 | 8·1585 | 5·27138 |  |
|  | Pre_NLR | 110 | 2·9726 | 2·02266 | <0·001 |
|  | NLR_8_21 | 110 | 7·9589 | 5·69414 |  |
|  | Pre_NLR | 112 | 3·0846 | 2·11733 | 0·019 |
|  | NLR_22_56 | 112 | 3·8631 | 3·07263 |  |
|  | **Pre_NLR** | **98** | **2·9493** | **2·03312** | **0·428** |
|  | **NLR_57_90** | **98** | **3·2726** | **3·47582** |  |
| LMR | Pre_LMR | 154 | 3·4443 | 1·69638 | <0·001 |
|  | Post_LMR | 154 | 1·8307 | 2·98153 |  |
|  | Pre_LMR | 156 | 3·4373 | 1·69966 | <0·001 |
|  | LMR_1_3 | 156 | 1·9390 | 1·18861 |  |
|  | Pre_LMR | 141 | 3·4517 | 1·55537 | <0·001 |
|  | LMR_4_7 | 141 | 2·0770 | 1·06396 |  |
|  | Pre_LMR | 110 | 3·2620 | 1·72841 | <0·001 |
|  | LMR_8_21 | 110 | 1·8956 | 0·94330 |  |
|  | **Pre_LMR** | **112** | **3·2875** | **1·78397** | **0·147** |
|  | **LMR_22_56** | **112** | **3·0477** | **1·45893** |  |
|  | **Pre_LMR** | **98** | **3·2831** | **1·68392** | **0·330** |
|  | **LMR_57_90** | **98** | **3·1221** | **1·63617** |  |
| Abbreviations: PLR, platelet-lymphocyte ratio; NLR, neutrophil-lymphocyte ratio; LMR, lymphocyte-monocyte ratio | | | | | |

**Supplementary Table S2.** Summary of the different treatment regimens for different tumour subtypes

|  |  | **Origin of Primary Tumour** | | | | | |
| --- | --- | --- | --- | --- | --- | --- | --- |
|  |  | **Colorectal, N= 161** | **Ovarian, N= 121** | **Appendiceal, N= 111** | **Primary Peritoneal, N= 26** | **Mesothelioma, N= 17** |  |
| **Neoadjuvant Chemotherapy** | Xelox | 3 |  | 1 |  |  |  |
|  | TS-1 and Oxaliplatin | 1 |  |  |  |  |  |
|  | Xeloda | 1 |  |  |  |  |  |
|  | FOLFOX | 1 |  | 1 |  |  |  |
|  | Paclitaxel + Carboplatin |  | 16 |  | 2 |  |  |
|  | Carboplatin |  | 1 |  |  |  |  |
|  | Unspecified |  | 1 |  |  |  |  |
|  | Paclitaxel + Carboplatin + Avastin |  |  |  | 1 |  |  |
| **Neoadjuvant Chemo-radiotherapy** | LCCRT | 1 |  |  |  |  |  |
|  | Xeloda + RT | 1 |  |  |  |  |  |
| **Definitive Chemotherapy** | Xeloda + Oxaliplatin | 1 |  |  |  |  |  |
|  | Xelox | 2 |  | 1 |  |  |  |
|  | Xelox + Xeloda |  |  | 1 |  |  |  |
|  | Paclitaxel + Carboplatin |  | 1 |  | 1 |  |  |
| **Definitive Chemo-radiotherapy** | Paclitaxel + Carboplatin |  | 1 |  |  |  |  |
|  | Capecitabine |  |  | 1 |  |  |  |
| **Adjuvant Chemotherapy** | Xelox + Xeloda | 8 |  |  |  |  |  |
|  | Xelox | 35 | 1 | 2 |  |  |  |
|  | Unspecified | 5 | 7 | 1 | 1 |  |  |
|  | 5FU + Oxaliplatin | 3 |  |  |  |  |  |
|  | Xeloda | 15 |  | 3 |  |  |  |
|  | Xelox + Avastin | 2 |  |  |  |  |  |
|  | Oxaliplatin | 1 |  |  |  |  |  |
|  | Xeloda + Oxaliplatin | 1 |  |  |  |  |  |
|  | Folfox | 4 |  | 1 |  |  |  |
|  | 5FU | 4 |  |  |  |  |  |
|  | CPT11 + FU + FA | 1 |  |  |  |  |  |
|  | Folfox + Avastin | 2 |  | 1 |  |  |  |
|  | Cape-Ox | 1 |  | 1 |  |  |  |
|  | Folfox + 5FU + Oxali + Bev | 1 |  |  |  |  |  |
|  | Avastin + CPT | 1 |  |  |  |  |  |
|  | Capecitabine + Erbitux + CI5FU + Oxaliplatin | 1 |  |  |  |  |  |
|  | Irinotecan + Cetuximab | 1 |  |  |  |  |  |
|  | Degramont 5FU | 1 |  |  |  |  |  |
|  | Folfiri + Avastin + Panitumumab + Folfox | 1 |  |  |  |  |  |
|  | Folfox + Bevacizumab | 1 |  |  |  |  |  |
|  | Oxaliplatin + Capecitabine + Bevacizumab + Fluorouracil + Cetuximab + Irinotecan | 1 |  |  |  |  |  |
|  | Paclitaxel + Carboplatin + Cisplatin + Avastin |  | 1 |  |  |  |  |
|  | Gem + CDDP IP + Paclitaxel + Carboplatin |  | 1 |  |  |  |  |
|  | Paclitaxel + Carboplatin |  | 50 |  |  |  |  |
|  | Paclitaxel + Carboplatin + CDDP |  | 1 |  |  |  |  |
|  | Gem + Paclitaxel + Carboplatin |  | 1 |  |  |  |  |
|  | CDDP IP + Paclitaxel |  | 1 |  |  |  |  |
|  | Paclitaxel + Carboplatin + Avastin |  | 3 |  | 3 |  |  |
|  | Gemcitabine + Cisplatin |  | 2 |  |  |  |  |
|  | Carboplatin |  | 5 |  |  |  |  |
|  | Cisplatin + Cyclophosphamide |  | 1 |  |  |  |  |
|  | Carboplatin + Avastin |  | 1 |  |  |  |  |
|  | Paclitaxel + Carboplatin + MoRab |  | 1 |  |  |  |  |
|  | Carboplatin + Bleomycin + Etoposide |  | 1 |  |  |  |  |
|  | Cisplatin + Doxorubicin + Gemcitabine + Cisplatin |  | 1 |  |  |  |  |
|  | MTX + VP16 |  | 1 |  |  |  |  |
|  | Paclitaxel + Carboplatin + Aromasin + MTX + CTX + Imquomod |  | 1 |  |  |  |  |
|  | Paclitaxel + Carboplatin + Caelyx |  | 1 |  |  |  |  |
|  | Paclitaxel + Carboplatin + gemcitabine + Taxotere + Avastin |  | 1 |  |  |  |  |
|  | Paclitaxel + Carboplatin + Gemcitabine |  | 1 |  |  |  |  |
|  | Yondelis + Caelyx + Paclitaxel + Gemcitabine + Cisplatin |  | 1 |  |  |  |  |
|  | Paclitaxel + Carboplatin + Bevacizumab |  |  |  | 1 |  |  |
|  | 5FU + FA |  |  | 1 |  |  |  |
|  | Anz + Carboplatin |  |  | 1 |  |  |  |
|  | Xeloda + TS-1 + Bevacizumab + CPT-11 + Mitomycin C + Ci5FU |  |  | 1 |  |  |  |
|  | Xelox + FOLFOX + Panitumumab |  |  | 1 |  |  |  |
|  | Xeloda + Carboplatin |  |  | 1 |  |  |  |
|  | Gemcitabine + Oxaliplatin + Xeloda |  |  | 1 |  |  |  |
|  | Xeloda + Capecitabine1 |  |  | 1 |  |  |  |
|  | 5FU + Leucovorin + Xeloda + Paclitaxel + Carboplatin |  |  | 1 |  |  |  |
|  | Taxane + Carboplatin |  |  |  |  |  |  |
|  | Cisplatin |  |  |  |  | 1 |  |
|  | Paclitaxel + Carboplatin + Alimta |  |  |  |  | 1 |  |
|  | Paclitaxel + Carboplatin + Alimta + CDDP |  |  |  |  | 2 |  |
| **Adjuvant Chemo-radiotherapy** | Unspecified | 1 |  |  |  |  |  |
|  | Xelox | 2 |  | 1 |  |  |  |
|  | Oxaliplatin + Capecitabine |  | 1 |  |  |  |  |

s


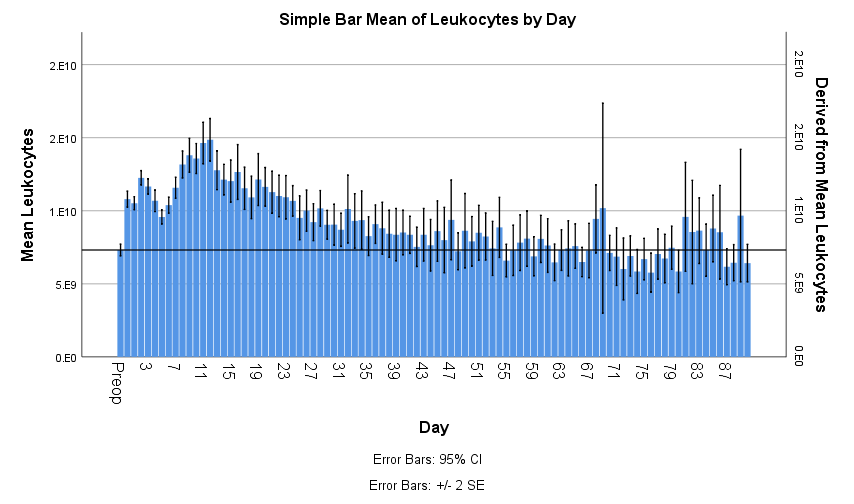

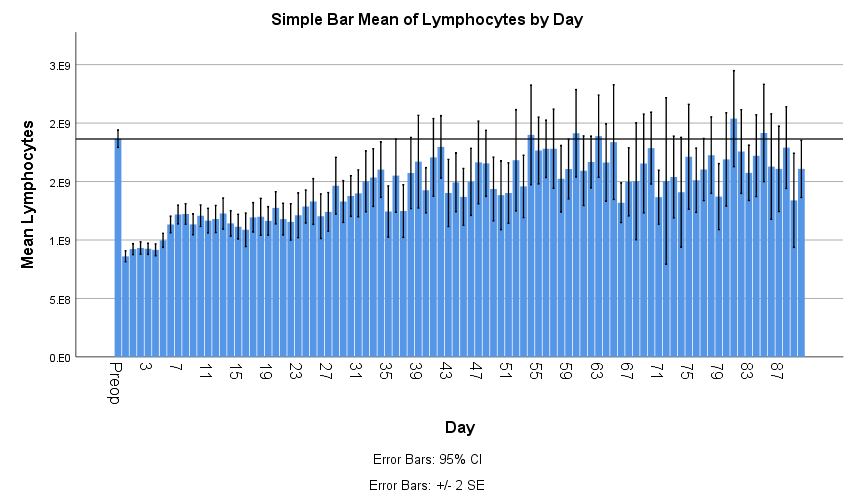

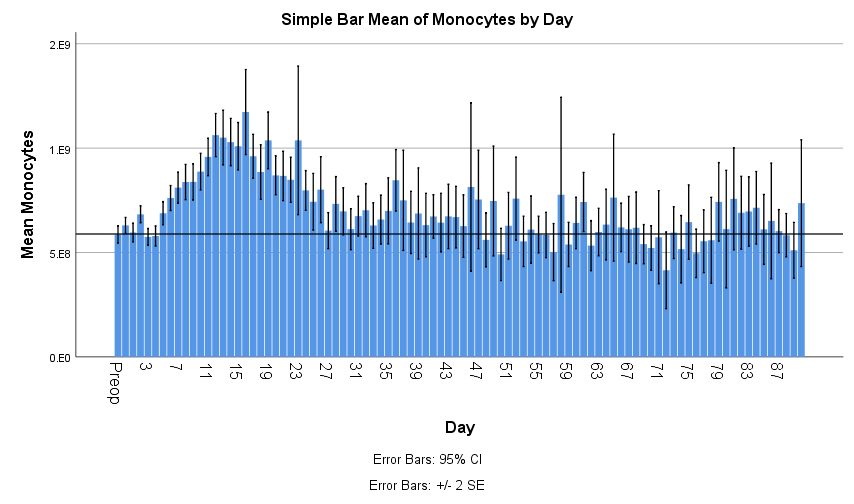

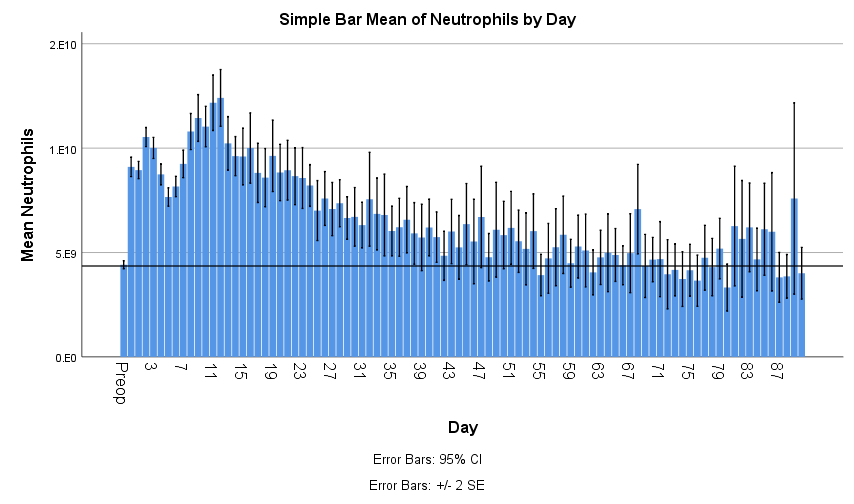

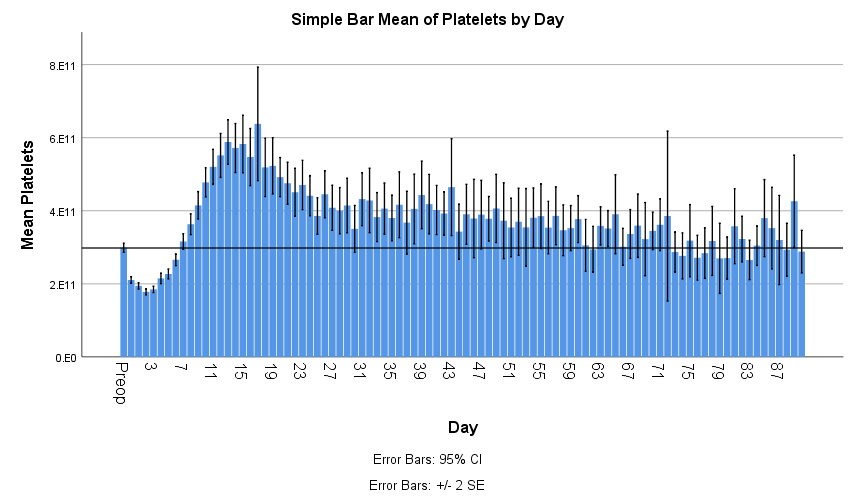


**Supplementary Figure S1:** Profile of immune cell response in patients across time (i.e. from pre-operation to postoperative day 90).

**Supplementary Figure S2:** Flowchart illustrating study population.


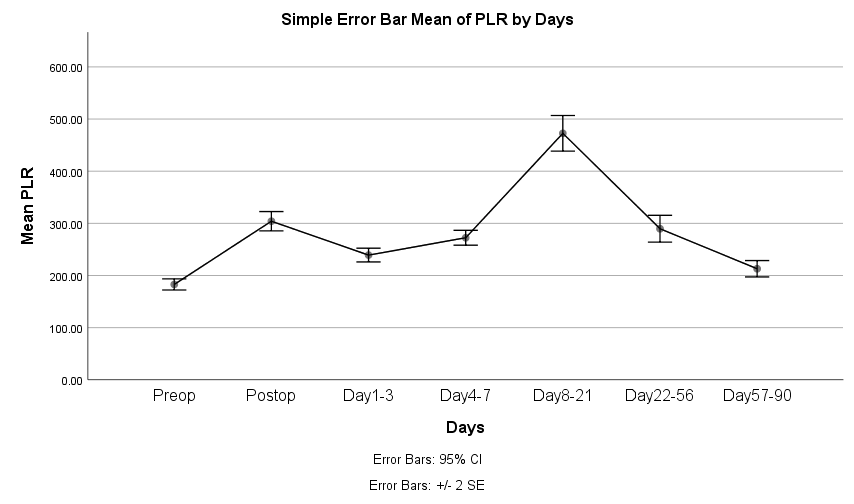

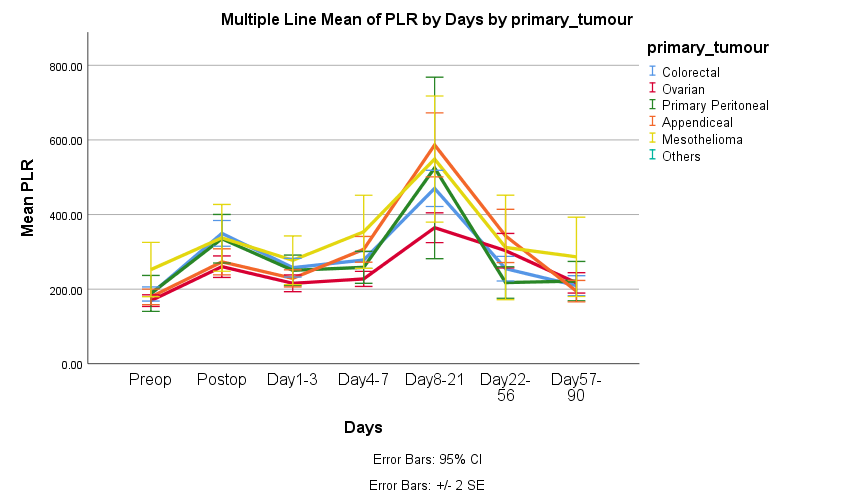

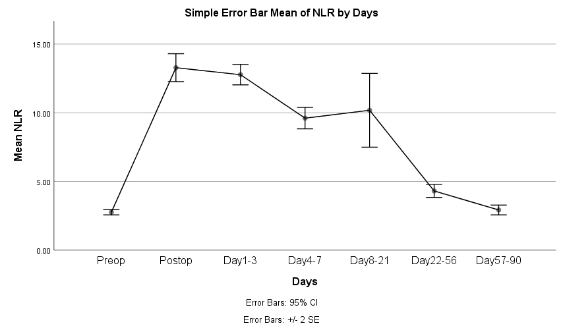

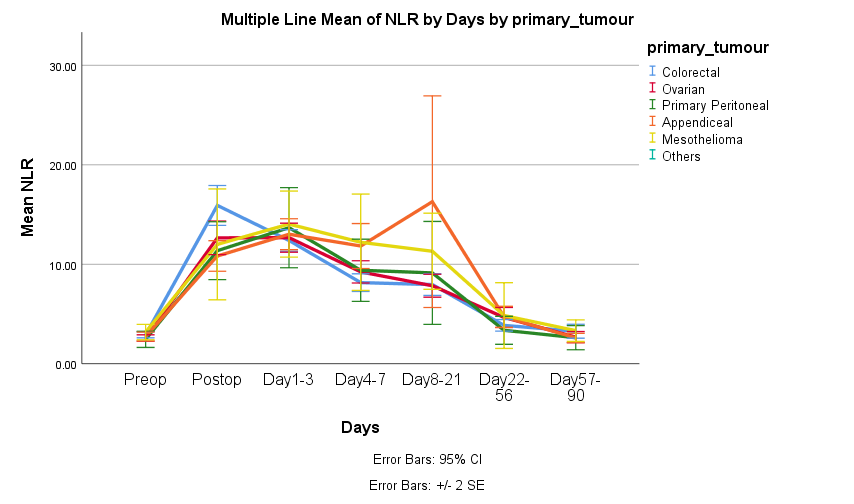

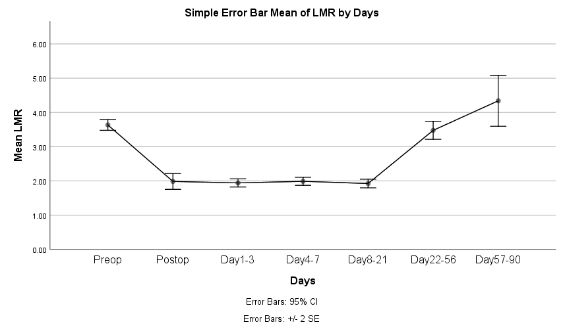

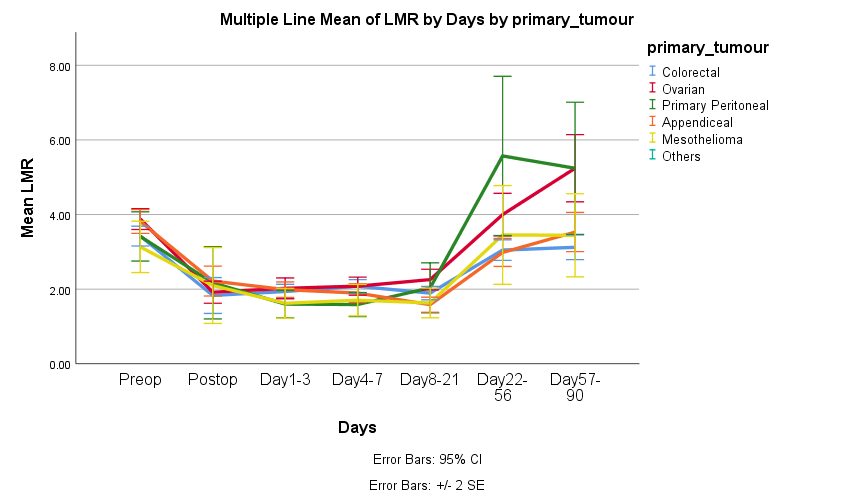


**Supplementary Figure S3:** Trend of PLR, NLR and LMR in patients across time (i.e. from pre-operation to postoperative day 90) by primary tumour type.


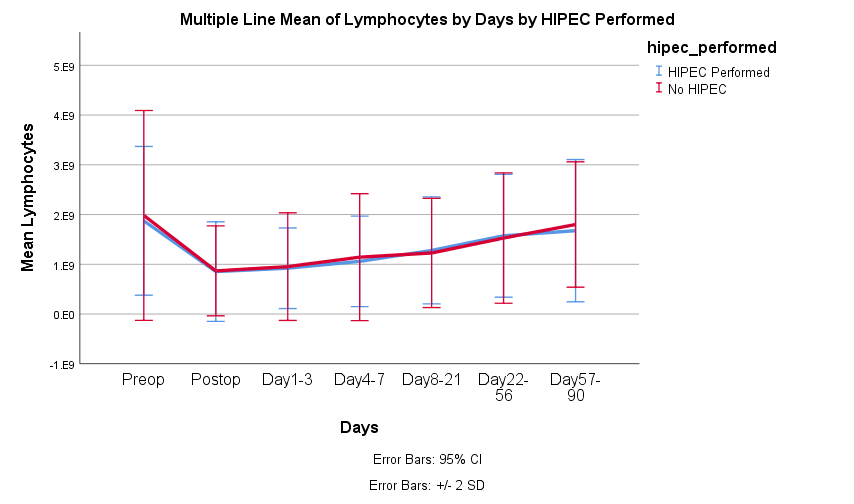

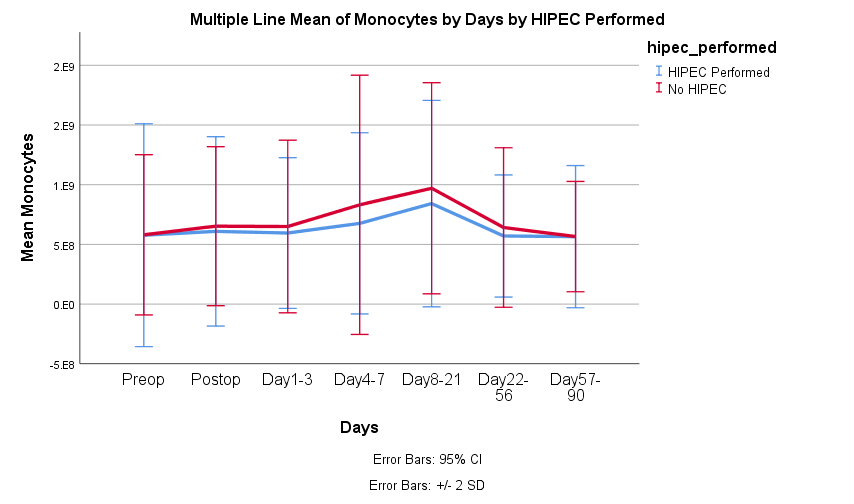

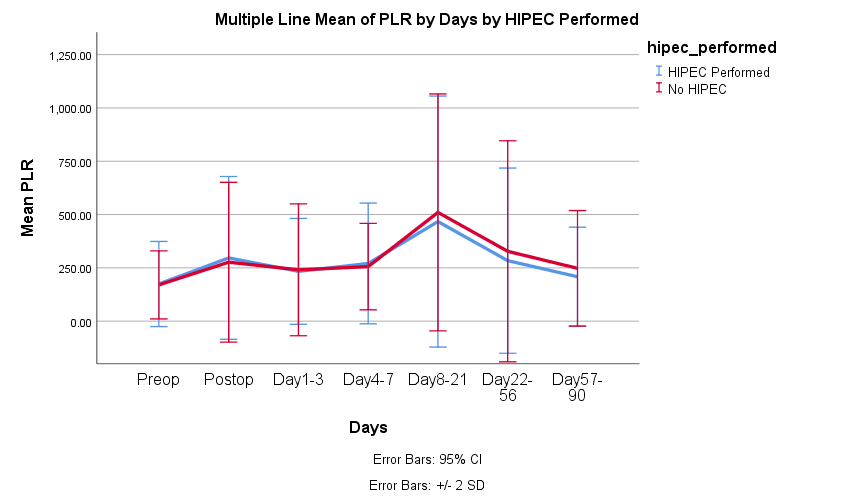

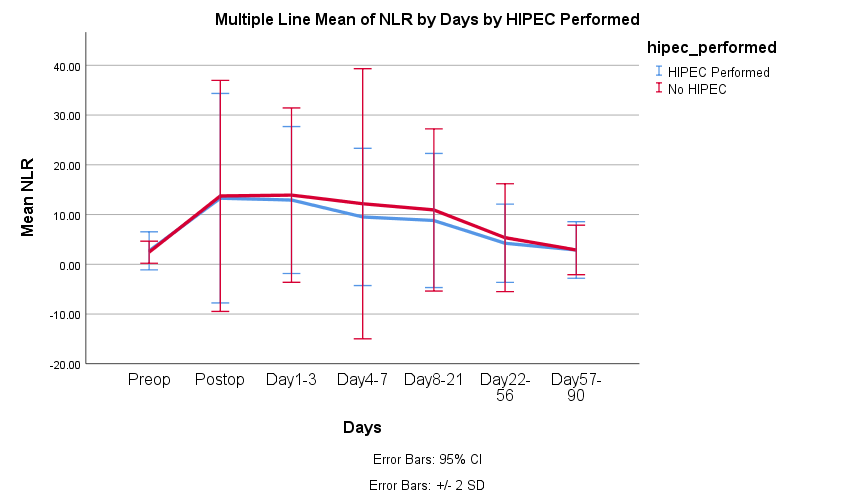

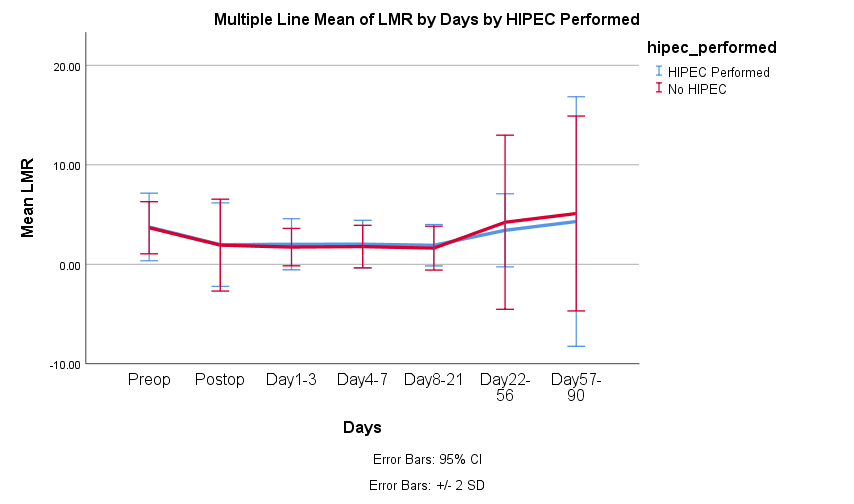

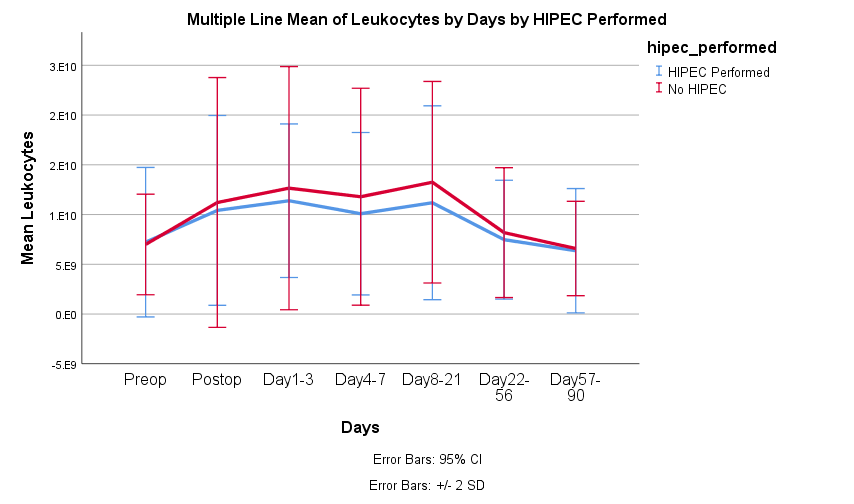


***HIPEC PerformedHIPEC Performed**


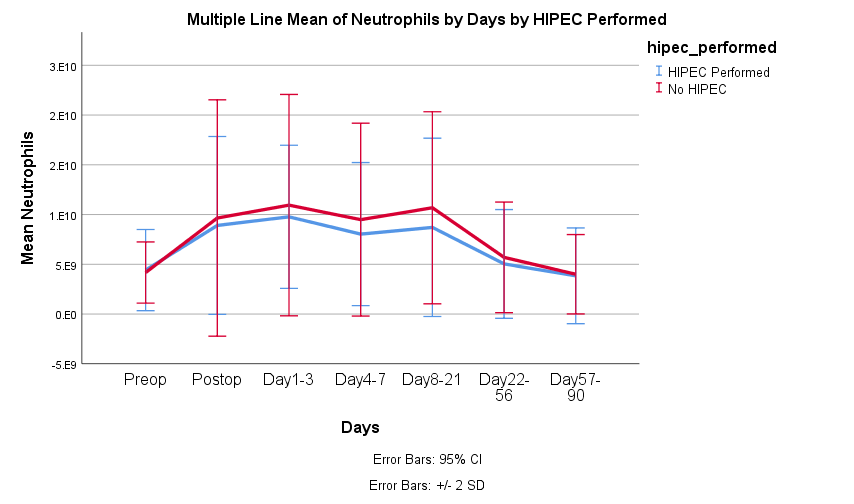


***HIPEC PerformedHIPEC Performed**


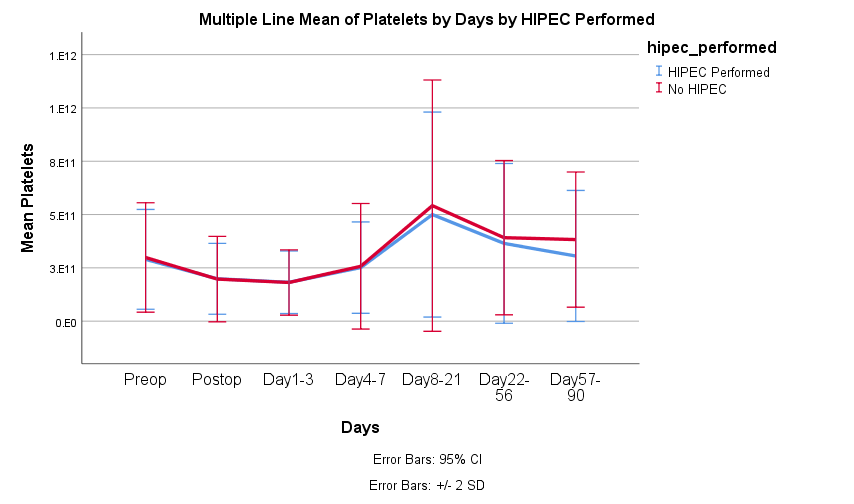


***HIPEC PerformedHIPEC Performed**

**Supplementary Figure S4:** Comparison of the profile of immune cell response in all peritoneal carcinomatosis patients across time (i.e. from pre-operation to postoperative day 90) based on whether they underwent HIPEC (n = 331) or no HIPEC (n = 39)

* denotes significance at *p* < 0.05 for Mann-Whitney U Test
